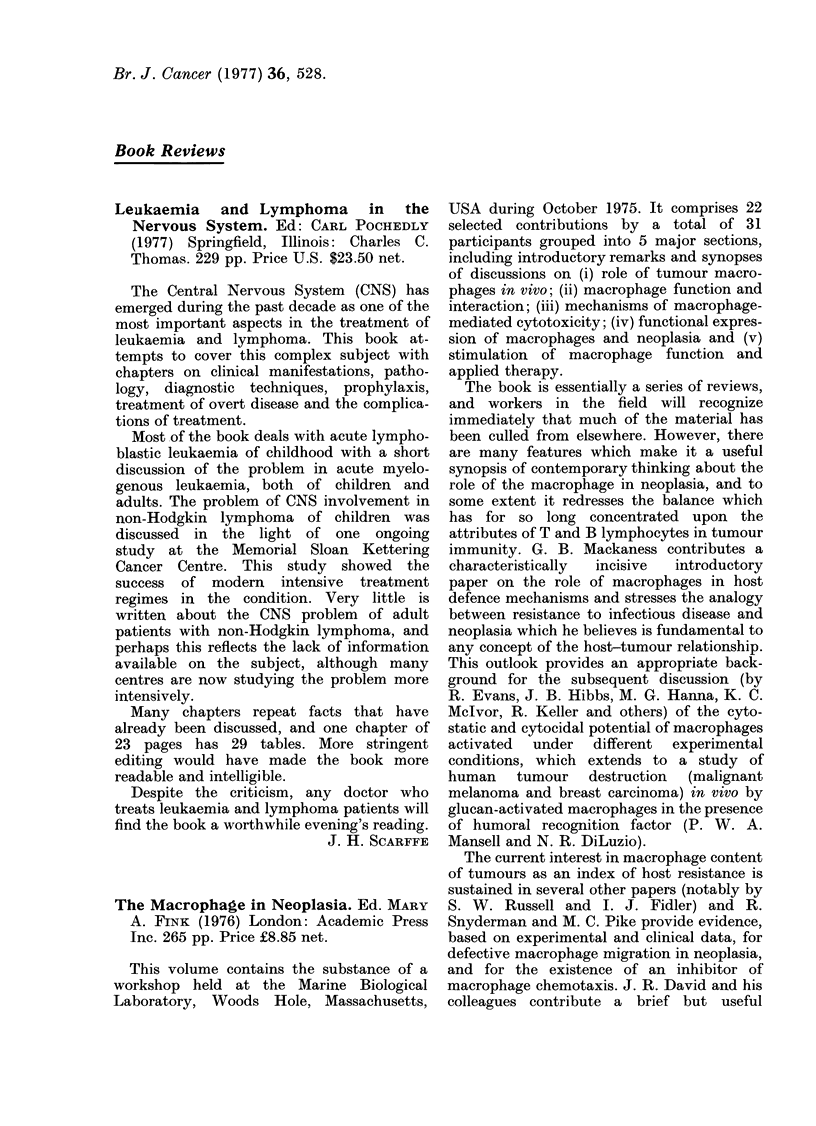# Leukaemia and Lymphoma in the Nervous System

**Published:** 1977-10

**Authors:** J. H. Scarffe


*Br. J. Cancer* (1977) **36**, 528.

### *Book Reviews*

**Leukaemia and Lymphoma in the Nervous System.** Ed: CARL POCHEDLY (1977) Springfield, Illinois: Charles C. Thomas. 229 pp. Price U.S. $23.50 net.

The Central Nervous System (CNS) has emerged during the past decade as one of the most important aspects in the treatment of leukaemia and lymphoma. This book attempts to cover this complex subject with chapters on clinical manifestations, pathology, diagnostic techniques, prophylaxis, treatment of overt disease and the complications of treatment.

Most of the book deals with acute lymphoblastic leukaemia of childhood with a short discussion of the problem in acute myelogenous leukaemia, both of children and adults. The problem of CNS involvement in non-Hodgkin lymphoma of children was discussed in the light of one ongoing study at the Memorial Sloan Kettering Cancer Centre. This study showed the success of modern intensive treatment regimes in the condition. Very little is written about the CNS problem of adult patients with non-Hodgkin lymphoma, and perhaps this reflects the lack of information available on the subject, although many centres are now studying the problem more intensively.

Many chapters repeat facts that have already been discussed, and one chapter of 23 pages has 29 tables. More stringent editing would have made the book more readable and intelligible.

Despite the criticism, any doctor who treats leukaemia and lymphoma patients will find the book a worthwhile evening's reading.

J. H. SCARFFE